# Supplementary material for: In-hospital mortality among immunosuppressed patients with COVID-19: Analysis from a national cohort in Spain
Source: PLoS One. 2021 Aug 3;16(8):e0255524. doi: 10.1371/journal.pone.0255524 (PMC8330927; doi:10.1371/journal.pone.0255524)
Supplement: S1 Table — (DOCX) [file pone.0255524.s001.docx]

S1 Table. Number of deaths (%), total number of patients, and crude and adjusted OR for death among immunosuppressed patients, patients with specific diseases or conditions (cancer [solid organ or haematologic], solid organ transplant or systemic autoimmune diseases), and patients receiving immune suppressive treatments prior to admission (systemic steroids, biological treatments, or immunosuppressors). All analyses have use non-immunosuppressed patients as reference category.

|  | Deaths: n (%) | N | OR (95% CI) | aOR* (95% CI) | p* |
| --- | --- | --- | --- | --- | --- |
| Non-IS | 2143 (19.3) | 11095 | 1 | 1 |  |
| IS | 661 (31.3) | 2111 | 1.90 (1.72-2.11) | 1.54 (1.36-1.73) | <0.001 |
| Patients with specific diseases and conditions | | | | | |
| All cancers (SO and H) | 465 (33.3) | 1398 | 2.08 (1.81-2.38) | 1.57 (1.36-1.82) | <0.001 |
| SO cancer | 343 (31.7) | 1081 | 1.94 (1.66-2.27) | 1.39 (1.18-1.65) | <0.001 |
| SO cancer with MT | 84 (30.4) | 276 | 1.82 (1.37-2.43) | 1.99 (1.42-2.78) | <0.001 |
| SO cancer, no MT | 259 (32.2) | 805 | 1.98 (1.63-2.41) | 1.25 (1.03-1.53) | 0.027 |
| Hematologic cancer | 139 (38.8) | 358 | 2.42 (1.92-3.05) | 2.38 (1.79-3.15) | <0.001 |
| Leukaemia | 66 (39.3) | 168 | 2.70 (1.89-3.84) | 2.09 (1.36-3.23) | 0.001 |
| Lymphoma | 77 (40.0) | 194 | 2.75 (2.16-3.51) | 2.81 (2.06-3.82) | <0.001 |
| Transplant | 57 (34.3) | 166 | 2.18 (1.60-2.99) | 2.70 (1.94-3.75) | <0.001 |
| Patients receiving immune suppressive treatments prior to admission | | | | | |
| Systemic steroids | 202 (35.4) | 570 | 2.29 (1.96-2.68) | 1.87 (1.55-2.27) | <0.001 |
| Biological treatment | 49 (26.8) | 183 | 1.52 (1.06-2.19) | 1.96 (1.65-2.33) | 0.001 |
| Immunosuppressors** | 109 (27.7) | 394 | 1.59 (1.27-1.99) | 1.81 (1.43-2.29) | <0.001 |

Non-IS: non-immunosuppressed patients. IS: immunosuppressed patients. SAID: systemic autoimmune diseases. OR: crude odds ratio. CI: confidence interval. aOR: adjusted odds ratio. SO: solid organ. H: haematological. MT: metastases.

*Adjusted for use of in-hospital steroid use, age, sex, level of dependency, smoking status, and comorbidities (arterial hypertension, chronic heart failure, chronic obstructive bronchopulmonary disease, asthma, dementia, moderate-severe chronic liver disease, moderate-severe chronic renal failure, and diabetes mellitus)

**Immunosuppressors include: azathioprine, methotrexate, tacrolimus, cyclophosphamide, mycophenolate, cyclosporin, rapamycin, and everolimus.
