# Supplementary material for: In-hospital mortality among immunosuppressed patients with COVID-19: Analysis from a national cohort in Spain
Source: PLoS One. 2021 Aug 3;16(8):e0255524. doi: 10.1371/journal.pone.0255524 (PMC8330927; doi:10.1371/journal.pone.0255524)
Supplement: Attachment — Submitted filename: PLOS ONE_Review_10May2021.docx [file pone.0255524.s003.docx]

**PLOS ONE**

**In-hospital mortality among immunosuppressed patients with COVID-19: analysis from a national cohort in Spain**

--Reviewer Comments—

1. Summary of Research

Suárez-García et al provided a detailed breakdown of characteristics of immunosuppressed (IS) patients hospitalized with COVID-19 and assessed the association between IS status and in-hospital mortality in this cross-sectional study of data from the SEMI-COVID-19 Network. This is the largest study of IS patients with COVID-19 to date, and it showed higher odds of mortality among those with IS compared to those without IS with differences by IS condition. The results add to the literature a more detailed picture of the characteristics and prognosis of those with IS who are hospitalized with COVID-19. The manuscript would benefit from some reorganization and clarification of the methods/results, clearer definitions of exposures and some outcomes, updates to the tables, including providing more thorough footnotes, and additional minor edits.

2. Major Improvements

- Exposure classification (Immunosuppressed). The authors state in the limitations that they don’t have data on HIV/AIDS, which is why those conditions were not included in the definition of Immunosuppressed (IS). Do the authors have data on the following conditions that could also be considered as a classification for IS: Stem cell transplantation, immunoglobulin deficiency, complement deficiency, asplenia, or cytopenias or other immunodeficiencies? If not, perhaps those could be included in the limitations.
- Exposure classification (Immunosuppressed). The authors classified those with systemic corticosteroid use as IS. Systemic corticosteroid use is common treatment for COVID-19 among hospitalized patients. Do the authors have data on route, duration, or dosing of steroid therapy that might have enable classification of whether steroids were immunosuppressive? Otherwise I might suggest classifying patients with only systemic steroids as non-IS, at least for the primary analysis. A secondary/sensitivity analysis could consider those patients as IS.
- Methods, Statistical Analysis: It is not clear how the authors modeled length of hospital stay (secondary outcome #2). Did they dichotomize the length of stay (e.g. 0-2 days versus 2+ days)? Did they consider using Cox proportional hazards to model time to discharge? Either way, the methods aren't clear on how this secondary outcome was assessed. In addition, the author did not present the results of this analysis, but they briefly include it in the discussion. The authors should clarify the methods and include the results of this analysis.
- Results: I recommend including the overall mortality and mortality by each stratum to the paragraph discussing the results from Table 1. I would also add row for mortality and other outcomes to table 1, both overall and by each stratum. Authors could add a column with overall study patients, and add rows for in-hospital mortality, ICU admission, and hospital length of stay.
- Results: I also recommend including subheadings within the results section for each of the primary and secondary analyses.

3. Minor Improvements

- Methods, Study Design. The authors state the study design is retrospective cohort; however, the study design is actually cross-sectional. The exposure and outcome data among the SEMI-COVID-19 cohort are ascertained at the same time for this study, although it uses data from a cohort.
- Methods, Patient Selection. The authors could consider adding a sentence or two describing the case definition criteria for the SEMI-COVID-19 Network and whether hospitalizations included in this study are laboratory-confirmed.
- Title. If the cases are laboratory-confirmed, perhaps change the title to “In-hospital mortality among immunosuppressed patients with laboratory-confirmed COVID-19: analysis from a national cohort in Spain”.
- Methods, Statistical Analysis: Perhaps the authors could state in the methods that the in-hospital complications are dichotomized (yes/no). E.g. on line 184, they could state "3. Dichotomized (yes/no) in-hospital complications (pneumonia, ARDS, etc...."
- Methods, Statistical Analysis: The authors should include how they defined “nosocomial pneumonia” on line 1984. Also, it is unclear why authors are only interested in nosocomial pneumonia rather than all types of pneumonia.
- Results: the authors cite a cutoff date for inclusion in the study of June 19th, but in the first line of the results it says "June 16th".
- Results (Table 2): I recommend editing the footnote to be a bit more specific or adding additional rows for units in the table explaining that under laboratory tests, the first value is a mean or median (?) and the value in parentheses is SD. It is unclear as is.
- Results: In the first paragraph following Table 3 on page 17, I recommend including the aOR's and 95% confidence intervals for the models within the text of the results section.
- Results: In the first paragraph following Table 4 starting on page 18, the sentences beginning with “In order to investigate” through “in addition to all other variables” is describing methods, and as such, should be moved to the methods section. Right now, the methods and results for this analysis are being combined in the results section.

4. Additional Suggestions

- Throughout the manuscript, the authors use the term “risk,” but since the model provides an estimate of the odds ratio, then the term “odds” is more accurate. The authors should consider changing the word “risk” to “odds” throughout the manuscript.
- I would present percentages with the n’s throughout the entire manuscript. The n’s are difficult to interpret.
  - All throughout the abstract, also include percentages following the n’s. (e.g. on line 90 “Among IS patients, a total of 166 (X.X%) had solid organ (SO) transplant...”)
  - In the manuscript, also include the percentages. (e.g. on line 228 “Among the IS patients, 166 (XX.X%) had SO transplant…”)
  - On line 233, authors report only percentages and not n’s. I would do both throughout the entire manuscript for consistency and clarity.
